# Effect of Microwave Annealing on the Sensing Characteristics of HfO_2_ Thin Film for High Sensitive pH-EGFET Sensor

**DOI:** 10.3390/mi14101854

**Published:** 2023-09-28

**Authors:** Siwei Cui, Hui Yang, Yifei Zhang, Xing Su, Dongping Wu

**Affiliations:** 1 State Key Laboratory of ASIC and System, Fudan University, Shanghai 200433, China; 18112020003@fudan.edu.cn (S.C.);; 2 School of Microelectronics, Fudan University, Shanghai 200433, China; 3 Institute of Photonic Chips, University of Shanghai for Science and Technology, Shanghai 200093, China

**Keywords:** hafnium oxide, EGFET pH sensor, microwave annealing, high sensitivity

## Abstract

Recently, certain challenges have persisted in PH sensor applications, especially when employing hafnium oxide (HfO_2_) thin films as sensing layers, where issues related to sensitivity, hysteresis, and long-term stability hamper performance. Microwave annealing (MWA) technology, as a promising solution for addressing these challenges, has gained significant attraction due to its unique advantages. In this article, the effects of microwave annealing (MWA) treatment on the sensing behaviors of Extended-Gate Field-Effect Transistors (EGFETs) utilizing HfO_2_ as a sensing film have been investigated for the first time. Various power levels of MWA treatment (1750 W/2100 W/2450 W) were selected to explore the optimal processing conditions. A thorough physical analysis was conducted to characterize the surface of the MWA-treated HfO_2_ sensing thin film using techniques such as X-ray photoelectron spectroscopy (XPS) and atomic force microscopy (AFM). Our findings reveal that MWA treatment effectively increased the surface sites (*Ns*) in the HfO_2_ sensing thin film, consequently leading to an increase in the pH sensitivity of EGFETs to 59.6 mV/pH, as well as a reduction in hysteresis and an enhancement in long-term stability. These results suggest that MWA offers a straightforward, energy-efficient method to enhance overall HfO_2_ sensing film performance in EGFETs, offering insights for HfO_2_ applications and broader microelectronics challenges.

## 1. Introduction

In 1970, the pioneering work of Bergveld led to the development of the ion-sensitive field-effect transistor (ISFET), marking a substantial milestone in the field of pH sensing and the measurement of ion concentrations [1,2]. Since then, numerous studies have been devoted to improving the sensing capabilities of the ISFET due to its inherent issues of low sensitivity and instability [3,4]. Given that ISFETs are directly integrated onto the FET electrode, they come with several disadvantages such as device instability, low current sensitivity, susceptibility to light interference, etc. [5]. Optical effects, hysteresis, and drift are the three non-ideal characteristics observed in ISFET-based pH sensors. To overcome these drawbacks, researchers introduced the Extended-Gate Field-Effect Transistor (EGFET), which has gained widespread popularity in biosensor applications [6,7,8,9,10]. EGFETs are favored for their long-term stability, insensitivity to light and temperature drift, and disposability. Functioning on the same working principles as ISFETs, EGFETs comprise a sensing structure and an independent MOSFET [6,11]. The sensing structure includes a sensing thin film in direct contact with the electrolyte, which is directly connected to the FET via a metal line. The sensing film, when exposed to an electrolyte solution, changes in response to variations in the H^+^ concentration of the tasted solution [11,12,13,14,15]. As a result, selecting an appropriate sensing film compatible with CMOS technology becomes crucial for achieving both the sensitivity and long-term stability of the sensor.

Sensitive materials have become the focal point of recent advancements in pH sensing technology, emphasizing miniaturization, affordability, convenience, and real-time sensing. In addition, the sensitivity [16] and corrosiveness [17] to alkali metal ions restrain the application of pH measurements in strongly acidic and alkaline solutions. In order to be compatible with advanced complementary metal oxide semiconductor (CMOS) technology, thin high-k dielectric films are promising candidates for the manufacturing of pH sensors. In Table 1, several high-k dielectric materials, such as SiO_2_, Si_3_N_4_, Al_2_O_3_, IGZO, and Ta_2_O_5_, are presented with relatively high sensitivity. However, these materials come with their own set of challenges. For example, SiO_2_ and Si_3_N_4_-gated ISFETs [18,19] exhibit non-uniform sensitivity responses to variations in pH levels. The Al_2_O_3_ layer [20,21,22] tends to detach after prolonged contact with the test liquid, while the IGZO layer [23,24] demonstrates pronounced susceptibility to corrosion in alkaline environments. Additionally, Ta_2_O_5_ [18,25] displays considerable sensitivity to light. These disadvantages impose limitations on their application in pH detection. On the other hand, ultra-thin hafnium oxide (HfO_2_) has attracted much attention due to its exceptional sensitivity and stability [26,27,28].

However, several instability phenomena arising from defects in the sensing film, such as charge traps, buried points, and slow reactions, hinder the application of EGFETs. Therefore, it is necessary to fabricate high-quality films with minimal defects to serve as sensing thin films. The commonly accepted sensing mechanism of a pH sensor could be explained with the site dissociation model, referring to the chemical reaction between free hydrogen ions in the electrolyte and amphoteric groups on the surface of the sensing thin film. Among these surface treatment methods, microwave annealing (MWA) treatment holds unique advantages, including reduced heat impact, as well as instantaneous and uniform heating. The ability of MWA to provide rapid and even heating holds significant promise, especially in the context of treating PH-sensitive films, offering a more controlled and precise treatment process.

In this article, the pH sensing characteristics of HfO_2_ sensing layers were investigated, where their response was examined through a series of experiments with various MWA treatment powers. These evaluations were carried out using an EGFET structure, with the HfO_2_ sensing layers being fabricated using atomic layer deposition (ALD). Notably, MWA treatment yielded significant improvements in sensing performance, particularly in enhancing the pH sensitivity to 59.6 mV/pH, close to the Nernst limit. The linearity of sensitivity exceeds 99.9%. MWA also improved long-term stability, with sensitivity only dropping to 5% during tests up to 20 days. Furthermore, a monotonical relationship between increased sensitivity and MWA treatment power was established. The annealing power of 2450 W was identified as optimal, delivering markedly improved pH sensitivity and long-term stability. The above results indicate that microwave annealing is an effective method to improve the sensing ability of HfO_2_-EGFET pH sensors. The researchers used X-ray photoelectron spectroscopy (XPS) and atomic force microscopy (AFM) to analyze the surface composition and morphology of HfO_2_ to explain the mechanism of MWA treatment. Our findings indicate that the increase in surface roughness following annealing may be the underlying reason for the enhanced pH response.

## 2. Materials and Methods

### 2.1. Fabrication Process 

**Figure 1:**
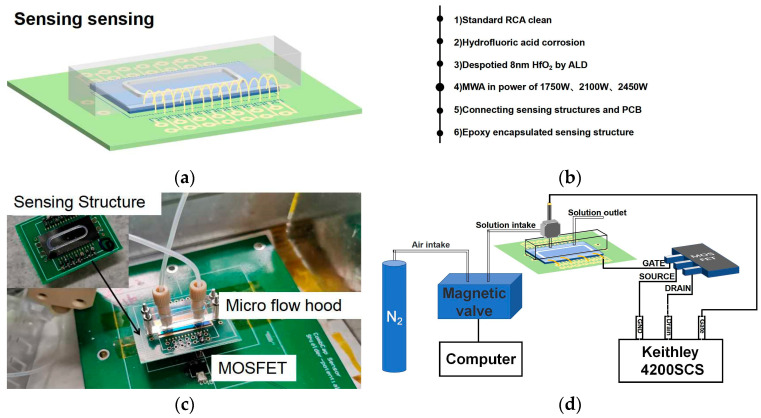
EGFET structures: (**a**) sensing structure; (**b**) preparation process of the entire sensing structure; (**c**) connection of sensing structure and MOSFET; (**d**) device diagram and electrical measurement system.

In this study, EGFET structures were fabricated on p^+^-doped Si substrate to investigate the pH-sensing properties of ALD-HfO_2_ films. As shown in Figure 1a, a gold electrode was deposited onto the sensing substrate, which is connected to the gold gate of the MOSFET. The electric potential of the sensing film surface will change the ion concentration when the sensing film makes contact with the electrolyte. Therefore, the electric potential variation at the electrolyte/insulator interface will be detected by the MOSFET. Figure 1b shows the entire preparation process of the entire sensing structure, where the HfO_2_ layer was deposited by ALD on a p^+^-doped Si wafer after performing RCA cleaning. Hf (NCH_3_C_2_H_5_)_4_ (TEMAH) was selected as the precursor and H_2_O was selected as an oxidant for the HfO_2_ thin films in an ALD reactor (BENEQ: TFS-200). The reactor chamber was evacuated by a vacuum pump to a base pressure of approximately 2 × 10^−2^ Pa at 300 °C. High purity N_2_ (99.999%) was introduced into the ALD chamber as both the carrier and purge gas during the film growth. An HfO_2_ sensing structure with a thickness of 8 nm was fabricated. After deposition, the samples were annealed using MWA in an N_2_ ambient (purity = 99.999%) for 600 s in power ranges of 1750 W, 2100 W, and 2450 W. Next, we used a 2% HF solution to etch the edges of the hafnium oxide in order to connect the silicon wafer to the epitaxial printed circuit board (PCB). 

The MWA treatment-sensing structure was pasted on the golden pad of the printed circuit board (PCB) with conductive tapes, and the golden pad and metal pins were connected by aluminum lines. A dispenser (TH-2004D-K, Tianhao, Zhejiang, China) was used to coat the surface of the sensing structure with epoxy resin adhesive along a custom elliptical path to provide a liquid pool with height. Poly (methyl methacrylate) (PMMA) was used to provide a closed induction chamber for the liquid reaction by pressing on a heightened epoxy resin. Finally, metal pins were used to connect the sensing structure to a commercial n-channel power MOSFET (MMFT960T1, ON Semiconductor Corporation, Phoenix, AZ, USA) gate, as shown in Figure 1c.

### 2.2. Physical and Electrical Measurements

The schematic diagram of the entire experiment is shown in Figure 1d. The microfluidic system was used to provide a stable environment for the measurements throughout the pH test. The Ag/AgCl electrode was inserted into the closed induction chamber filled with a pH buffer solution as a reference electrode, and the standard pH buffer solution (Reagecon, Shannon, Ireland) was passed into the closed induction chamber for pH measurement. It is worth noting that all the samples were immersed in the buffer solution (pH 7) for 30 min to stabilize before the first test. In this experiment, a Keithley 4200-SCS semiconductor parameter analyzer (Keithley, Cleveland, OH, USA) was used to test the electrochemical characteristics of the HfO_2_ -EGFET pH sensors. In the steady-state measurement, the source-drain voltage (V_ds_) was kept at 0.1 V and a reference voltage (V_M_) was swept one cycle from 0 V to 5 V at 0.01 V intervals; this was applied to the Ag/AgCl reference electrode. The source-drain current (I_ds_) was measured. In the real-time measurement conditions, the device was operated in the liner region by setting the reference voltage V_M_ to 3.8 V. 

The I_ds_ was measured as the pH in the loop changed from pH 4 to pH 10 and back. Each pH-stabilizing fluid flow was controlled for 25 s by means of a solenoid valve, and the pH was changed after a constant 30 s. The sensing behaviors such as sensitivity, hysteresis, and stability of the devices were continuously monitored over 20 days. The surface condition of the material was analyzed by XPS and AFM. The surface content was analyzed by a Kratos AXIS Ultra DLD XPS (Kratos, Kyoto, Japan) using an Al Ka X-ray source (1486.6 eV). The Bruker Dimension Icon AFM (Bruker, Karlsruhe, Germany) was used to determine the surface morphology of the thin films over a scan area of 5 × 5 µm^2^.

## 3. Results and Discussion 

### 3.1. Sensing Thin Film Surface Analysis 

**Figure 2:**
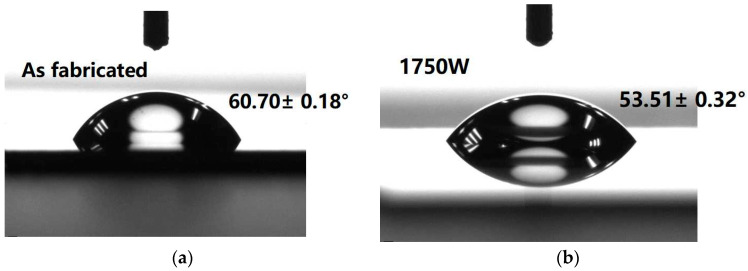

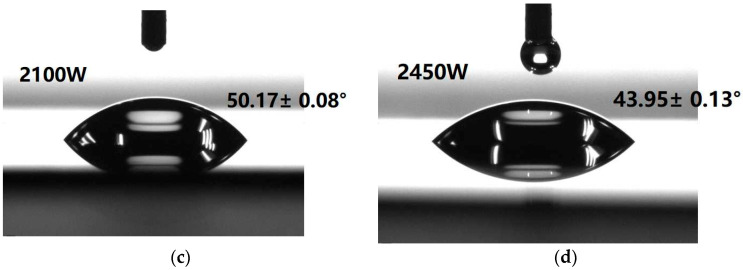
The evolution of the water contact angle as a result of the treatments achieved on the surfaces of the HfO_2_ films. (**a**) without microwave annealing; (**b**) 1750 W; (**c**) 2100 W; (**d**) 2450 W.

A wettability study was used to characterize the HfO_2_ surface by measuring the water contact angle, both before and after MWA. Figure 2 shows the evolution of the water contact angle as a result of the treatments achieved on the surfaces of the HfO_2_ films. Contact angles of 60.6 ± 0.2°, 52.9 ± 0.3°, 50.2 ± 0.1°, and 41.2 ± 0.1° were measured, showing a slight enhancement in the hydrophobic character of the HfO_2_ corresponding to a MWA of 0 W, 1750 W, 2100 W, and 2450 W, respectively. After treating the HfO_2_ surface with MWA, the contact angles exhibited a significant decrease of 32%. As previously reported by Braik et al. [27,28], this transformation rendered the HfO_2_ highly hydrophilic. 

**Figure 3:**
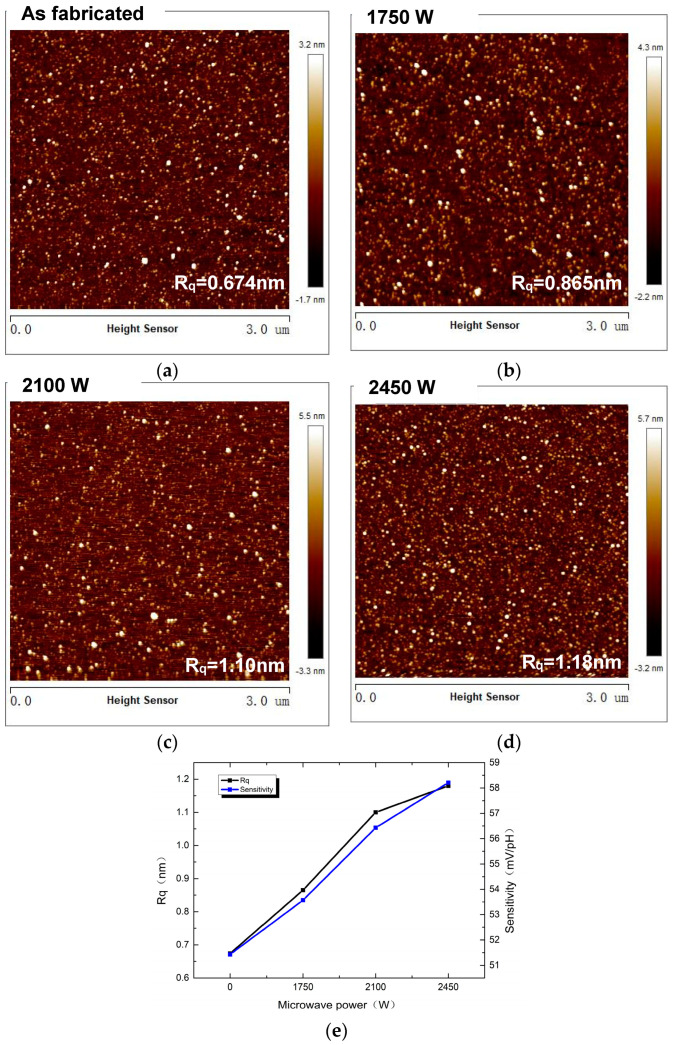
(**a–d**) show the morphologies of HfO_2_ films with the 0 W, 1750 W, 2100 W, and 2450 W conditions; (**e**) the relationship between sensitivity and surface roughness.

In our experiment, an AFM image was used to investigate the surface morphologies of the HfO_2_ films. For comparison, Figure 3a–d show the morphologies of the HfO_2_ films with the 0 W, 1750 W, 2100 W, and 2450 W conditions, respectively. The HfO_2_ films exhibited a columnar microstructure. Notably, under the 2450 W condition, the columnar grain size was found to be larger compared to the conditions at 0 W, 1750 W, and 2100 W. Additionally, the root mean square (Rq) roughness values for the films, corresponding to microwave-assisted (MWA) powers of 0 W, 1750 W, 2100 W, and 2450 W, were measured at 0.674 nm, 0.865 nm, 1.10 nm, and 1.18 nm, respectively. As the MWA power increased, the surface roughness of the HfO_2_ film gradually increased. The 2450 W condition showed the roughest surface. From the above-mentioned results, we assumed that the improvement in roughness of the film occurred as the MWA power increased. Actually, the surface morphologies are relevant to the increase in *Ns*. The observed transition of the film microstructures from a homogeneous surface to a rough surface may be ascribed to the greater quantity of -OH groups in the HfO_2_ film to transform its structure, as shown in Figure 3e.

**Figure 4:**
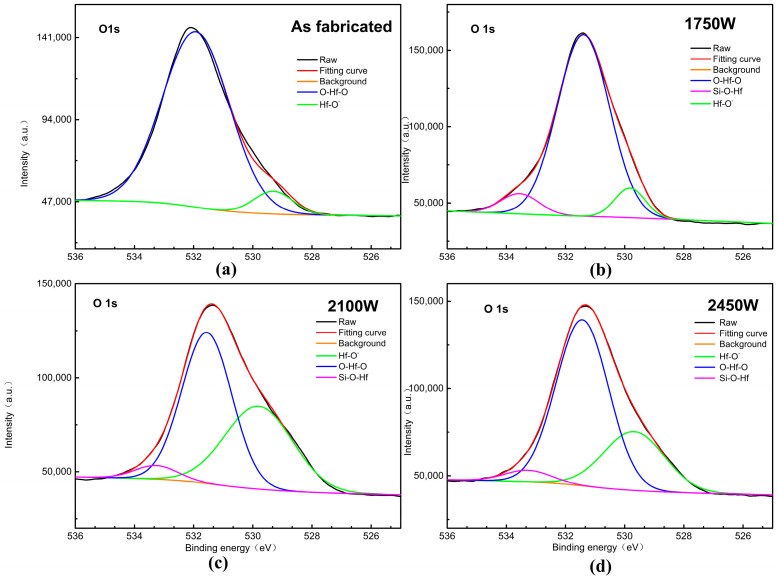
O 1s XPS spectra of the HfO_2_ films performed (**a**) without microwave annealing; (**b**) at 1750 W; (**c**) 2100 W; (**d**) 2450 W power.

Apart from the film morphology and structure, the chemical compositions of the HfO_2_ films after MWA at three different powers were examined using XPS. Figure 4a–d present the O 1s XPS spectra of the HfO_2_ films performed at the three MWA powers, respectively. The O 1s peak is de-convoluted into two components: one at 529.8 eV, corresponding to the Hf-OH bonds, and the other at 532.1 eV, corresponding to the Hf-O-Hf bonds. 

Table 2 provides detailed composition information for the HfO_2_ films. It can be seen that the area of the Hf-OH shows a growing trend with the increase in MWA power. The –OH groups near the surface acted as buried sites resulting in hysteresis, so hysteresis increased with the increase in the –OH groups after the MWA treatment [21]. The red line and black line were expressed as the fitted data and experimental data, respectively. The O 1s core-level spectra of the HfO_2_ films with multiplet splitting are shown in Figure 4. We define the peak at 529.8 eV as the peak of Hf-O, with the peak variation attributed to the partial Si-O production; 532.1 eV is the peak of Hf-O-Si, which decreases in area as the annealing power increases, and therefore the silicate content decreases. Finally, the area of the 529.2 eV Hf-O peak varies as a function of annealing temperature and is maximal at 2100 W, which means that the oxygen containing groups have the highest content at this time.

### 3.2. EGFETs Sensing Behaviours

**Figure 5:**
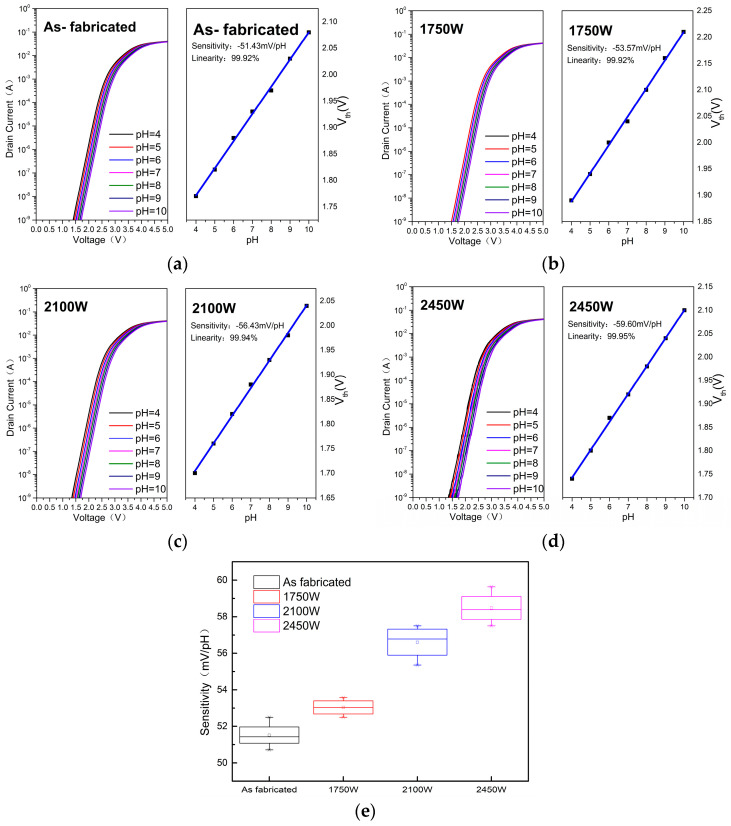
Transfer and sensitivity characteristics of HfO_2_-EGFET pH sensors over different MWA powers: (**a**) as fabricated; (**b**) 1750 W; (**c**) 2100 W; (**d**) 2450 W; (**e**) the statistical sensitivity $\mu_{FE}$ the HfO_2_-EGFET pH sensors with different MWA powers.

Figure 5 illustrates the transfer and sensitivity characteristics of the HfO_2_-EGFET pH sensors over different MWA power treatments. As shown in Figure 5a–d, the threshold voltage (V_t_) is positively correlated with pH, reflecting that the surface potential of the sensing film increases with the hydrogen ion concentration. The threshold voltage of the EGFETs was defined as V_t_ when I_ds_ reached 1 × 10^−7^ A, and the sensitivity was obtained by linearly fitting the V_t_ with different pH values, as shown in the right diagram of Figure 5a–d. For each annealing power, more than three samples were tested, and the obtained sensitivity was averaged and calculated. The relationship between the average sensitivity and the MWA processing power is shown in Figure 5e. It can be seen that with the increase in the annealing power of MWA, the sensitivity of the HfO_2_-EGFET pH sensor will gradually increase.

According to past articles, the sensitivity of EGFTE can be expressed as:

(1) $$\mathrm{Sensitivity}=2.3\frac{kT}{q}\alpha ,\;\mathrm{with}\;\alpha =\frac{1}{1+\frac{2.3kTC_{di}}{q^{2}\beta_{int}}}$$

In Equation (1), *k* refers to the Boltzmann’s constant, *T* refers to the absolute temperature, *q* refers to the elementary, *β_int_* refers to the intrinsic buffer capacity, and *C_di_* refers to the differential capacitance. It can be seen that the pH sensitivity at a fixed temperature is influenced by buffer capacity *β_int_* and differential capacitance *C_di_*. This article focuses on the buffering ability *β_int_* to buffer small pH changes on the surface of the sensing thin film, which depends entirely on the intrinsic properties of the sensing material such as the surface site number *Ns* and the equilibrium constants of *Ka* and *Kb*. *β_int_* can be expresses as:

(2) $$\beta_{int}=\frac{2q^{2}N_{s}{\left(\frac{K_{a}}{K_{b}}\right)}^{1/2}}{kTC_{di}}$$

where, when the material is identified as HfO_2_, the reaction constant and the dissociation constant associated with the acid–base equilibrium are definite values that can be set to a constant value = 16 μF/cm^2^, in order for the sensitivity of the ISFET device to increase with the increase in the surface site density *Ns*. When the surface amphiphilic group reacts with H^+^, the surface sites exist in the form of Hf-O, Hf-OH^2+^, and Hf-OH, and the number of surface sites *Ns* is the sum of these three sites. Based on Figure 5, Ns as well as the sensitivity showed a significant increase with the increasing power of the MWA treatment, showing a monotonic increasing trend [20]. In addition, with the increase in the MWA power, the particles inside the film get more energy and a stronger lateral movement ability, and the refinement of the spikes on the surface of the film is more obvious, which leads to the increase in roughness, which could increase sensitivity [29]. 

**Figure 6:**
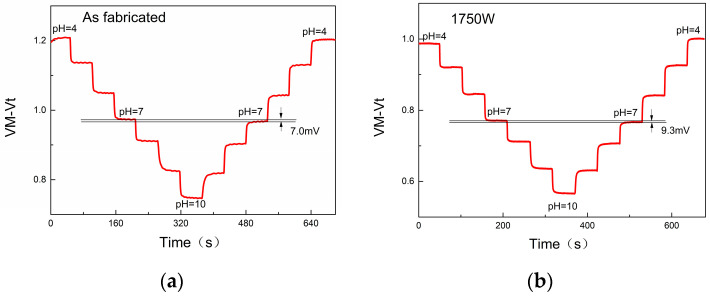

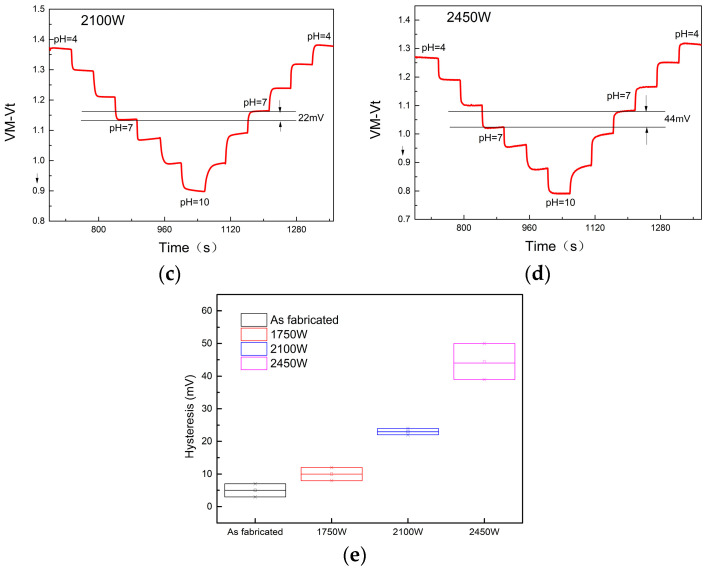
(**a**–**d**) Hysteresis curves of HfO_2_-EGFETs over different MWA powers: (**a**) as fabricated; (**b**) at 1750 W; (**c**) 2100 W; (**d**) 2450 W; (**e**) the statistical hysteresis $\mu_{FE}$ the HfO_2_-EGFET pH sensors with different MWA powers.

To acquire further understanding of the HfO_2_-EGFET pH sensor’s sensing behaviours, it was tested in real-time in this experiment by cycling the buffer solution from pH 10 to pH 4, and back. During this process, we maintained a constant gate bias voltage (V_M_) of MOSFET at 3.8 V to ensure the device works in the linear region. The shift of the threshold voltage (V_M_ − V_t_) was calculated using the formula V_M_ − V_t_ = I_ds_/gm, where gm represents the transconductance. The phenomenon of hysteresis is that V_M_ − V_t_ is not consistent for the same pH during multiple cycles of the test. Here, the hysteresis width (△V_t_) is defined as the difference in V_M_ − V_t_ between the pH 7 of the neighboring loops (as shown in Figure 6a–d). The hysteresis of the devices without microwave annealing treatment was about 3 mV at a minimum, while the hysteresis showed a noticeable increase with the increase in the MWA power. In this case, the average hysteresis value of multiple devices after microwave annealing treatment at 2450 W for 600 s was 44 mV. Such hysteresis was attributed to defects in the sensing membrane. Typically, the device’s response is primarily attributed to the interfacial reaction occurring between the solution and the sensitive membrane. This interaction encompasses both rapid and gradual response processes. The fast response is mainly caused by the reaction between the surface amphiphilic groups and the hydrogen ions in the electrolyte; the slow response mainly has an overall response between the hydrated ions in the buried sites below the sensing membrane. After MWA, the oxygen-containing group Hf-OH on the surface of the sensitive membrane increases and the surface defects become larger, resulting in an observable increase in immediate hysteresis. 

**Figure 7:**
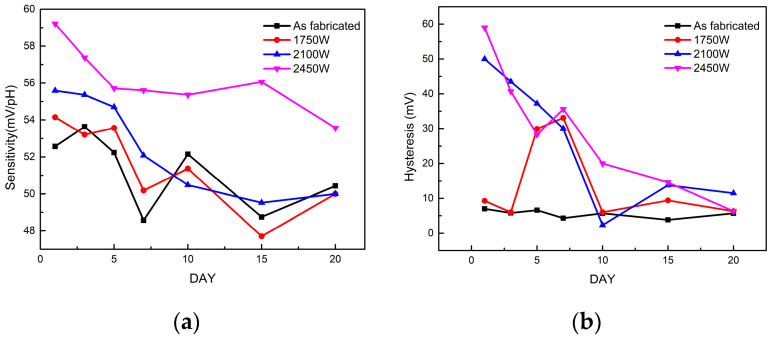
Comparisons of sensitivity (**a**) and hysteresis (**b**) characteristics over the 20-day period.

To investigate the long-term stability of MWA-treated devices, sensing behaviours of multiple sets of devices were continuously evaluated over a 20-day duration. The comparisons of sensitivity and hysteresis characteristics over the 20-day period are shown in Figure 7. It was found that the sensitivity and hysteresis of the devices decreased with increasing time, and basically reached stability after about 6 days. The sensitivity of the unannealed device decreases from the initial 51.43 mV/pH to 48.45 mV/pH after 20 days, while the hysteresis basically maintains a fluctuation range of 3 mV, ranging from 7 mV to 5.7 mV. In contrast, the device annealed at 2450 W exhibited a decrease from the initial 59.60 mV/pH to 54.57 mV/pH after 20 days, which is greater than the 20-day sensitivity of the other three groups of devices, and also greater than the unannealed device, with only a 6.25% reduction. The hysteresis, on the other hand, decreases from an initial 44.2 mV on day 1, which decreases to 16.2 mV after day 5, and subsequently fluctuates around 10 mV, ultimately decreasing to 6.3 mV after 20 days.

## 4. Conclusions

The effects of MWA treatment on the sensing behaviours of EGFETs utilizing HfO_2_ sensing films have been studied. The investigation focused on how MWA treatment influenced the sensing performance of HfO_2_ sensing film-based EGFETs. The AFM and XPS results reveal that the HfO_2_ sensing film exhibits increased surface roughness and a higher concentration of OH-related atomic groups on the surface. As a result, the EGFET demonstrates excellent sensing performance, including a notably high sensitivity up to 59.6 mV/pH and long-term stability with a fluctuation of less than 5% over a 20-day period.

## Figures and Tables

**Table 1:** Performance comparison of pH sensor devices with different sensitive films.

Sensing Layer	Sensitivity (mV/pH)	Linearity (%)	Reference
SiO_2_	40	-	[18,19]
Si_3_N_4_	53~55	-	[18,19]
Al_2_O_3_	56~59	>99	[20,21,22]
IGZO	41.5–57.4	99.4	[23,24]
Ta_2_O_5_	54–58	-	[18,25]
HfO_2_	59.6	>99.9	This work

**Table 2:** Elemental composition of the surface and bulk based on XPS measurement.

Treatment	Hf-OH (Area%)	Hf-O-Hf (Area%)	Hf-OH/Hf-O-Hf
NO MWA	11.82	88.18	13.40%
1750 W MWA	20.76	79.24	26.20%
2100 W MWA	28.09	71.91	39.06%
2450 W MWA	20.95	79.05	26.49%

## Data Availability

Not applicable.
